# Text-Guided Image Editing Based on Post Score for Gaining Attention on Social Media

**DOI:** 10.3390/s24030921

**Published:** 2024-01-31

**Authors:** Yuto Watanabe, Ren Togo, Keisuke Maeda, Takahiro Ogawa, Miki Haseyama

**Affiliations:** 1 Graduate School of Information Science and Technology, Hokkaido University, N-14, W-9, Kita-ku, Sapporo 060-0814, Hokkaido, Japan; y_watanabe@lmd.ist.hokudai.ac.jp; 2 Faculty of Information Science and Technology, Hokkaido University, N-14, W-9, Kita-ku, Sapporo 060-0814, Hokkaido, Japan; togo@lmd.ist.hokudai.ac.jp (R.T.); maeda@lmd.ist.hokudai.ac.jp (K.M.); ogawa@lmd.ist.hokudai.ac.jp (T.O.)

**Keywords:** text-guided image editing, diffusion model, posted image editing, post score, social media marketing

## Abstract

Text-guided image editing has been highlighted in the fields of computer vision and natural language processing in recent years. The approach takes an image and text prompt as input and aims to edit the image in accordance with the text prompt while preserving text-unrelated regions. The results of text-guided image editing differ depending on the way the text prompt is represented, even if it has the same meaning. It is up to the user to decide which result best matches the intended use of the edited image. This paper assumes a situation in which edited images are posted to social media and proposes a novel text-guided image editing method to help the edited images gain attention from a greater audience. In the proposed method, we apply the pre-trained text-guided image editing method and obtain multiple edited images from the multiple text prompts generated from a large language model. The proposed method leverages the novel model that predicts post scores representing engagement rates and selects one image that will gain the most attention from the audience on social media among these edited images. Subject experiments on a dataset of real Instagram posts demonstrate that the edited images of the proposed method accurately reflect the content of the text prompts and provide a positive impression to the audience on social media compared to those of previous text-guided image editing methods.

## 1. Introduction

With significant advances in Internet technologies, social media has become an important part of our daily lives. Social media provides a wide range of interactions such as communicating with other users, seeking and providing news, buying and selling products, and advertising. The number of users using social media is expected to increase further in the future [1], and interactions within social media have the potential to have a significant impact on society. Instagram, which has grown particularly rapidly in recent years, has more than 1.3 billion monthly active users in 2023 [2]. Instagram is a platform specialized in posting images and videos and allows users to “like” and comment on posts. The extraordinary success of Instagram confirms a recent report by Pew Research Center (https://www.pewresearch.org/ (accessed on 20 November 2023)) that photos and videos have become the primary social currency online [3]. In other words, images posted on Instagram have significant worth. To enhance the worth, there are more opportunities to edit images, and research on automatic image editing is becoming important [4,5,6].

Representative automatic image editing approaches include image colorization [7], image inpainting [8], and style transfer [9], which are expected to eliminate the tedious human work involved in image editing. These approaches are usually constructed for one-pattern transformations. For example, the approach of style transfer automatically transforms a style in an input image to a specific artist’s style, and there are limitations in transforming it to different artist’s styles using one model and in reflecting a user’s request into the edited image. To address these problems, several user-friendly approaches, named text-guided image editing, have been proposed [10,11,12,13,14,15]. Given an input image and a text prompt describing the contents of image editing from a user, text-guided image editing aims to edit the text-related region in accordance with the text prompt and maintain text-unrelated regions of the input image. With the great success of generative models such as StyleGAN [16] and the diffusion model [17] in recent years, text-guided image editing has become a hot topic.

**Figure 1:**
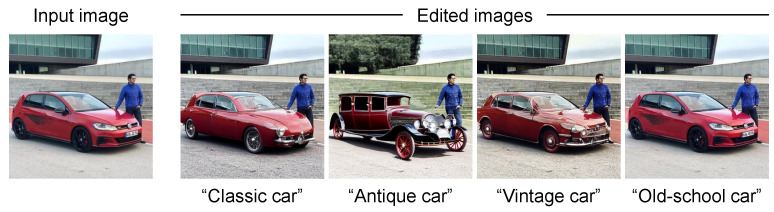
Example of different results of text-guided image editing depending on how the text prompts are given, even if they have the same meaning. The edited images are generated from Instruct-Pix2Pix [15] in accordance with the text prompts below each edited image. Note that this image was posted by the username @autogefuehl on Instagram, which is included in the Instagram influencer dataset [18].

**Figure 2:**
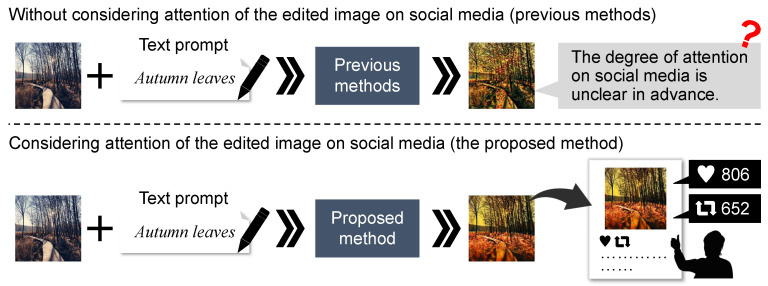
The difference between the proposed and previous text-guided image editing methods. In the upper case, although the image editing is performed in accordance with the text, it is unclear how much attention the edited image receives when it is posted on social media. In the lower case, the edited image, which is generated based on the text-guided image editing method considering the attention on social media in advance, will be displayed to a greater audience.

While text-guided image editing based on the diffusion model achieves particularly high performance, the user must expend effort devising how to give the text prompt to obtain the desired result. Specifically, as shown in Figure 1, the results of text-guided image editing differ depending on the way the text prompt is represented, even if it has the same meaning. It is up to the user to decide which result best matches the intended use of the edited image. When the intended use of the edited image is to post to social media, it is difficult for the user without knowledge of social media marketing to decide which result will gain attention from a greater audience. As shown in the upper case of Figure 2, it is unclear how much attention the result of the previous text-guided image editing method would gain when it is posted on social media. Moreover, as shown in the lower case of Figure 2, by performing text-guided image editing while considering the attention on social media in advance, the edited image can be displayed to a greater audience. From the above, it is necessary to construct a text-guided image editing method that can generate an edited image to gain attention on social media.

Here, there is much research on post and influencer classifications for social media marketing [18,19,20,21]. In post classification, several works [18,19] focus on the categories (e.g., fashion, travel, and food) and the virality (a situation in which a post gets more interactions than others on Twitter (currently X) and thus gains the attention from a relatively large number of users [19]) of posts on social media. In particular, the research classifying the virality of posts is useful in selecting edited images for posting on social media from the multiple results of text-guided image editing. However, since the conventional method [19] predicts virality only from posted text, it is impossible to predict the attention of edited images on social media in advance. Therefore, it is necessary to construct a new model that predicts in advance the attention from the audience on social media based on the posted image in addition to the posted text.

In light of the above, we propose novel text-guided image editing considering the response in social media in this paper. The goal of the proposed method is to provide the user with images that are edited in accordance with the text prompt and will gain attention from the greater audience on social media. Here, the degree of attention from the audience is defined as the engagement rate calculated from the number of likes and comments on a post. The key idea of the proposed method is to newly introduce a model to predict post scores representing engagement rates on social media from posted images and text, thereby generating edited images that gain attention from the greater audience. To construct the novel model, based on previous works [22,23] that analyzed the relationship between the content or aesthetic of posted images and the engagement rate from the perspective of computer vision, the proposed method focuses on aesthetics and categories of the posted images in addition to features of posted images and texts and calculates post scores. Then, the proposed method obtains several other expressions similar to the text prompt given by the user, based on a large language model that has attracted much attention in recent years. We apply a pre-trained text-guided image editing method and generate edited images from each of these several text prompts. Using multiple text prompts increases the possibility of obtaining edited images that gain attention from a greater audience on social media while performing image editing desired by the user. Among these, by leveraging the novel model that predicts post scores representing engagement rates, we finally select the edited images that will gain more attention from the audience on social media. To the best of our knowledge, this is the first text-guided image editing method considering the response from the audience on social media. The proposed method can provide users with edited images that have the potential to obtain the highest engagement rate and is expected to reduce the burden of creating posts for users without knowledge of social media marketing.

The rest of this paper is organized as follows. We introduce related works on social media marketing, image–text matching, and text-guided image editing in Section 2. In Section 3, we then explain the proposed method that performs text-guided image editing considering the response in social media. In Section 4, as a preliminary validation, we verify the accuracy of the proposed model to predict post scores. Section 5 demonstrates extensive experimental results for verifying the effectiveness of the proposed method. Finally, we conclude our work in Section 6.

## 2. Related Work

### 2.1. Social Media Marketing

Social media marketing is defined as the usage of social media platforms to promote products and services [24]. As social media marketing, influencer and post analyses has been conducted. To effectively find influencers who can have a positive impact on a product or service of a company, there has been much research on classifying influencers according to various aspects. Specifically, Liu et al. [25] classified influencers into three categories (i.e., emerging influencers, holding influencers, and vanishing influencers) by considering temporal changes in user trust networks. Using a massive dataset crawled from Instagram, Kim et al. [18] proposed a multimodal deep learning model to classify influencers into specific categories (e.g., fashion, travel, and beauty). These studies can clarify the potential influence and specialty categories of influencers and help companies decide which influencers to hire for promotions. Furthermore, there is research analyzing the social impact of the content on social media [26,27]. It is important to predict the popularity of posts by focusing on viral posts that influence social, economic, and political outcomes on Twitter (currently X). Rameez et al. [19] constructed a model based on machine learning to predict the virality from the post text and other information (e.g., the numbers of Hashtags, mentions, and followers). While the previous work [19] only focused on the text contained in the post, we calculate the post score on social media through a collaborative analysis of the posted image and the text in this paper.

### 2.2. Image–Text Matching

To analyze social media marketing based on machine learning, a collaborative analysis of vision and language plays a significantly important role. In the field of machine learning, other various tasks such as image captioning [28], text-to-image synthesis [29], cross-modal text–image retrieval [30], visual question answering [31], and referring image segmentation [32] have also benefited from image–text matching. To perform the collaborative analysis of vision and language, there are several studies [33,34] that aim to construct a common space that represents the highest similarity between the feature vectors of an image and its corresponding text. In previous works, convolution and recurrent-based neural networks such as CNNs and long-short term memory [35] have been applied as feature extractors in each modality. With the remarkable success of Transformer [36], contrastive language-image pre-training (CLIP) [37] has taken over its place and is playing an essential role in the field of the image–text matching. Since CLIP is trained on 400 million text–image pairs collected from a variety of public sources on the Internet, its primary strength is the extremely powerful representational capabilities acquired through training. This strength enables state-of-the-art zero-shot image classification on a variety of datasets. Shen et al. reported that CLIP provides significant benefits for downstream tasks, not only for image classification [38]. By employing CLIP as a feature extractor for posted images and texts, it is expected to acquire feature representations useful for calculating the post score.

### 2.3. Text-Guided Image Editing

To gain interest from a greater audience on social media, posted images tend to be edited, and research on automatic image editing is becoming extremely important [4,5,6]. From the perspective of less burden in image editing and easier reflection on user intentions, text-guided image editing has become an important topic. The goal of text-guided image editing is to edit the text-related region in accordance with the text prompt and preserve text-unrelated regions. The research on text-guided image editing was rapidly accelerated by the emergence of the generative adversarial network (GAN) [39]. Approaches to GAN-based text-guided image editing can be divided into two categories: (1) the approaches [10,11,40,41,42,43] utilizing a unique network with a single or multi-stage architecture, and (2) the approaches [12,13,44,45,46] leveraging the representation capabilities of a pretrained StyleGAN [16,47,48]. In approach (1), some studies [40,41] have applied an encoder–decoder architecture and successfully generated 64×64 resolution edited images on datasets such as Oxford-102 flower [49] and Caltech-UCSD Birds [50]. To generate high-resolution edited images on complex image dataset such as MSCOCO [51], several studies [10,11,42,43] construct a multi-stage architecture with a generator and discriminator at each stage. Three stages are trained at the same time, and progressively generate edited images of three different resolutions, i.e., 64×64→128×128→256×256. In approach (2), with the remarkable success of StyleGAN, the flexibility and quality of image editing have been dramatically improved in various classes, such as human and animal faces, churches, and cars. Several works [16,52] have reported that the immediate latent spaces W and W+ of StyleGAN are suitable for mixing images and semantic editing of images. Therefore, numerous works [12,13,44,45,46] leverage the representation capabilities of the pretrained StyleGAN and proposed novel image editing techniques. Furthermore, the success of the diffusion model [17] has brought significant benefits to the research field of text-guided image editing.

Unlike StyleGAN-based approaches, several studies [14,15] have taken advantage of the generative power provided by the diffusion model and realized a versatile text-guided image editing with no restrictions on the class of the edited object. The remarkable performance of recent generative models promises to further enhance the performance of text-guided image editing. However, there are still few approaches that consider the intended use of the edited image, and this paper proposes novel text-guided image editing considering the response in social media in this paper.

## 3. Proposed Text-Guided Image Editing Based on Post Score for Gaining Attention on Social Media

**Figure 3:**
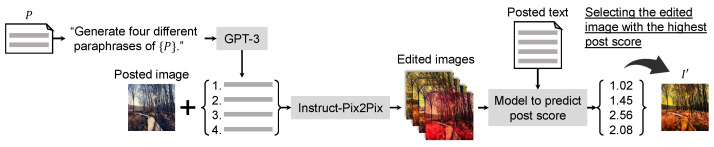
Overview of the proposed method. By applying Instruct-Pix2Pix [15], the proposed method generates four edited images using the posted image and four paraphrased texts obtained from GPT-3 [53]. Finally, the edited image with the highest post score is selected. The detail of the model to predict post scores is shown in Figure 4.

We show the architecture of the proposed method in Figure 3. In the proposed method, we first obtain four paraphrase expressions for the text prompt *P*. Then, the proposed method applies the pre-trained text-guided image editing method (i.e., Instruct-Pix2Pix) and generates four edited images. Finally, we leverage the proposed model to predict the post score and select the edited image $I^{\prime }$ with the highest post score from all results of the text-guided image editing method.

**Figure 4:**
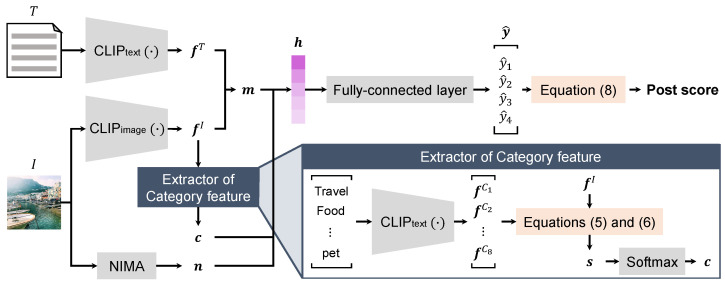
Details of the proposed model to predict post score. To calculate the post score, the proposed model predicts the class probability of the engagement rate using three integrated features h.

In the proposed model, we calculate the post score representing the engagement rate on social media from the posted image and text, as shown in Figure 4. Specifically, based on previous works [22,23] that analyzed the relationship between the content or aesthetic of posted images and the engagement rate, we focus on aesthetics and categories of the posted images in addition to features of posted images and texts and calculate post scores. We describe the details of the proposed model to predict post scores in Section 3.1. Then, the overall flow of the proposed method applying that model is described in Section 3.2.

### 3.1. Calculation of Post Score Representing Engagement Rate on Social Media

This section explains the proposed model that takes an image *I* and a text *T* as inputs and predicts a post score representing the engagement rate when they are posted on social media. As shown in Figure 4, to predict the post score, the proposed model uses three features (1) a multimodal feature extracted from the image *I* and text *T*, (2) an aesthetics feature of the image *I*, and (3) a category feature (e.g., fashion, travel, and food).

#### 3.1.1. Calculation of Post Score Using Multiple Features

To obtain the multimodal feature, we utilize contrastive language-image pre-training (CLIP) [37]. CLIP includes two neural networks of an image encoder ${\mathrm{CLIP}}_{\mathrm{image}}\left(\cdot \right)$ and a text encoder ${\mathrm{CLIP}}_{\mathrm{text}}\left(\cdot \right)$. CLIP is trained on a very large image–text pair dataset, thus extracting image and text features in highly expressive spaces. The proposed method obtains features $f^{I}\in R^{D}$ and $f^{T}\in R^{D}$ extracted from the image *I* and text *T* in the CLIP space as follows:

(1) $$\begin{array}{c} f^{I}={\mathrm{CLIP}}_{\mathrm{image}}\left(I\right), \end{array}$$

(2) $$\begin{array}{c} f^{T}={\mathrm{CLIP}}_{\mathrm{text}}\left(T\right), \end{array}$$

After deriving the image and text features, we concatenate these features and calculate the final multimodal feature $m\in R^{2D}$ as follows:

(3) $$\begin{array}{c} m=[f^{I};f^{T}]. \end{array}$$

This multimodal feature m is an important factor in predicting post scores because it provides a comprehensive representation of the image *I* and text *T* contained in a post.

With a previous work [23] that clears the relationship between the aesthetic of posted images and the engagement rates, we calculate an aesthetics feature of the image *I* to predict post scores. To calculate the aesthetics feature, we apply a neural image assessment (NIMA) model [54]. NIMA can predict the distribution of human opinion scores for image aesthetics using convolutional neural networks. The NIMA model takes an image as input and outputs a 10-dimensional softmaxed probability distribution representing the quality score. We use this distribution, which is output by inputting the image *I* into the NIMA model, as the aesthetic feature $n\in R^{10}$ in the proposed model to predict the post score. This feature n can represent the aesthetics of the image *I*, which potentially has a significant impact on the overall quality of the posts.

Considering the relationship between post categories and the engagement rates [22], we additionally focus on a category feature. Following [18], we distinguish eight categories of posts: beauty, family, fashion, fitness, food, interior, pet, and travel. However, since manually labeling posts into those eight categories requires a great deal of effort, the proposed model leverages the power of CLIP. Specifically, we first calculate the text features of the eight texts ${\left\{C_{i}\right\}}_{i=1}^{8}$ representing the category names in the CLIP space as follows:

(4) $$\begin{array}{c} {\left\{f^{C_{i}}\right\}}_{i=1}^{8}={\left\{{\mathrm{CLIP}}_{\mathrm{text}}\left(C_{i}\right)\right\}}_{i=1}^{8}. \end{array}$$

The proposed model calculates the cosine similarity $s_{i}$ between the text feature $f^{C_{i}}$ and the image feature $f^{I}$ obtained in Equation (1) as follows:

(5) $$\begin{array}{c} s_{i}=\frac{f^{I}\cdot f^{C_{i}}}{\left|\right|f^{I}{\left|\right|}_{2}\left|\right|f^{C_{i}}{\left|\right|}_{2}}. \end{array}$$

We obtain a vector s representing the category of the image *I* by concatenating the calculated cosine similarities ${\left\{s_{i}\right\}}_{i=1}^{8}$ as follows:

(6) $$\begin{array}{c} s=[s_{1};s_{2};\ldots ;s_{8}]. \end{array}$$

The proposed model finally obtains the category feature $c\in R^{8}$ by applying the softmax function to the vector s. The feature c can represent the category to which the image *I* belongs without manual labeling.

The proposed model predicts the class of the engagement rate based on the three calculated features m, n, and c. Specifically, we use the three features and first calculate the concatenated feature $h\in R^{2D+18}$ as follows:

(7) $$\begin{array}{c} h=[m;n;c]. \end{array}$$

This concatenated feature h can consider the aesthetics and categories of the image *I* while comprehensively representing the image *I* and text *T* contained in the post. The feature h is passed through a fully connected layer to obtain the class distribution $\hat{y}$ of the engagement rate. By using $\hat{y}$, the proposed model calculates the post score as follows:

(8) $$\begin{array}{c} \mathrm{Post}\;\mathrm{score}=\sum_{k=1}^{K}\left(k-1\right)\times {\hat{y}}_{k}, \end{array}$$

where ${\hat{y}}_{k}$ is the *k*th elements of the predicted class distribution $\hat{y}$, and *K* is the number of classes for engagement rates. The more skewed the distribution is toward classes with high engagement rates, the higher the post score.

#### 3.1.2. Loss Function

To predict the class distribution of the engagement rate, we apply cross entropy [55] as a loss function and treat the classification task for the class of the engagement rate. The loss function L is described as follows:

(9) $$\begin{array}{c} L=-\sum_{k=1}^{K}y_{k}\log{\hat{y}}_{k}, \end{array}$$

where $y_{k}$ is the *k*th elements of the ground truth class distribution y. By minimizing the loss calculated from this function L, it is possible to predict the class distribution of the engagement rate given the image *I* and text *T* as inputs.

### 3.2. Editing of Post Image Based on Post Score

To obtain multiple results of text-guided image editing, the proposed method first leverages a recently proposed large language model (i.e., GPT-3 [53]). We create the sentence “Generate four different paraphrases of {P}”. using the text prompt *P* to be used for image editing. The proposed method can obtain four representations similar to the text prompt *P* by giving the created sentence to the large language model. Using multiple text prompts increases the possibility of obtaining edited images that gain attention from a greater audience on social media while performing image editing desired by the user. The proposed method uses the four representations similar to the text prompt *P* and generates four edited images based on the text-guided image editing method [15].

To select the edited images that will receive the attention from a greater audience on social media, we apply the proposed model to predict post scores constructed in Section 3.1. The proposed method inputs the text to be posted and the four edited images, respectively, into that model and obtains four post scores. Finally, the proposed method selects the edited image $I^{\prime }$ with the highest post score from all edited images. From the above, it is possible to generate the image $I^{\prime }$ that is performed text-guided image editing and will gain attention on social media. By using the proposed method, the burden of using text-guided image editing can be reduced for users without knowledge of social media marketing.

## 4. Preliminary Validation

In this section, as a preliminary validation, we verify the accuracy of the proposed model that predicts post scores representing engagement rates on social media from the image and text. Section 4.1 and Section 4.2 provide validation settings and results, respectively.

### 4.1. Validation Settings

In the proposed model, we applied the image encoder based on vision transformer-B/32 [56] and text encoder based on Transformer [36] provided by CLIP [37]. These encoders extracted 512-dimensional (i.e., *D* = 512) image and text features. To calculate an aesthetic feature, the proposed model used the NIMA model [54] pre-trained on the aesthetic visual analysis (AVA) dataset [57]. During the training of the proposed model, we only trained the parameters of the fully connected layer while freezing the parameters of CLIP and the NIMA model.

As a dataset, we used the Instagram influencer dataset [18] which includes 10,180,500 Instagram posts with its engagement rate. To treat the classification task for the engagement rate, the dataset was divided into four classes (i.e., *K* = 4) as shown in Table 1.

Through the ablation study, we verified the accuracy of the proposed model. Specifically, we adopted the following four comparative models.

- CM1: the model that does not use the text feature.
- CM2: the model that does not use the aesthetic feature.
- CM3: the model that does not use the category feature.
- CM4: the method that does not use all features except an image feature.

As with the proposed model, these comparative models were trained for 50 epochs using the Adam optimizer [58] with a learning rate of 0.0001. Note that we applied an early stopping based on the accuracy of the validation set. By comparing the proposed model with these comparative models, we validate the effectiveness of predicting post scores based on three features (i.e., a multimodal feature, an aesthetic feature, and a category feature).

To evaluate the prediction performance, we used Accuracy and F-measure, which is the metric calculated as the harmonic mean of Recall and Precision defined as follows:

(10) $$\begin{array}{ccc} \mathrm{Accuracy} & = & \frac{\mathrm{Number}\;\mathrm{of}\;\mathrm{correctly}\;\mathrm{predicted}\;\mathrm{posts}}{\mathrm{Number}\;\mathrm{of}\;\mathrm{all}\;\mathrm{posts}}, \end{array}$$

(11) $$\begin{array}{ccc} \mathrm{F-measure} & = & \frac{2\times \mathrm{Recall}\times \mathrm{Precision}}{\mathrm{Recall}+\mathrm{Precision}}, \end{array}$$

where

(12) $$\begin{array}{ccc} \mathrm{Recall} & = & \frac{\mathrm{Number}\;\mathrm{of}\;\mathrm{correctly}\;\mathrm{predicted}\;\mathrm{posts}\;\mathrm{in}\;\mathrm{each}\;\mathrm{class}}{\mathrm{Number}\;\mathrm{of}\;\mathrm{correct}\;\mathrm{posts}\;\mathrm{in}\;\mathrm{each}\;\mathrm{class}}, \end{array}$$

(13) $$\begin{array}{ccc} \mathrm{Precision} & = & \frac{\mathrm{Number}\;\mathrm{of}\;\mathrm{correctly}\;\mathrm{predicted}\;\mathrm{posts}\;\mathrm{in}\;\mathrm{each}\;\mathrm{class}}{\mathrm{Number}\;\mathrm{of}\;\mathrm{all}\;\mathrm{posts}\;\mathrm{predicted}\;\mathrm{into}\;\mathrm{each}\;\mathrm{class}}. \end{array}$$

### 4.2. Accuracy of Proposed Model to Predict Post Scores

Table 2 shows the results of Accuracy and F-measure based on the proposed and comparative models in predicting classes for engagement rates. As shown in Table 2, the proposed model achieved the highest Accuracy and F-measure compared with the four comparative models. Specifically, the values of Accuracy and F-measure of CM1 and CM4, which use either posted image or text features, were lower compared to the other models. These results confirm the effectiveness of using posted images and texts collaboratively. The results of CM2 were lower than those of CM3, indicating that category features, rather than aesthetic features, are more significant in predicting classes of engagement rates. However, both results of CM2 and CM3 were lower than the proposed model using all features, indicating that category and aesthetic features contributed to the improvement in the performance to predict classes of engagement rates. Also, it can be seen that F-measure is equal to Accuracy in almost all cases. Since the equilibrium data shown in Table 1 was used in this experiment, the values of “true positive” and “true negative” in calculating F-measure and Accuracy were relatively equal, which led to these results. The values of Accuracy and F-measure seem low for the classification task, but comparable values have been shown in a similar study [19].

**Figure 5:**
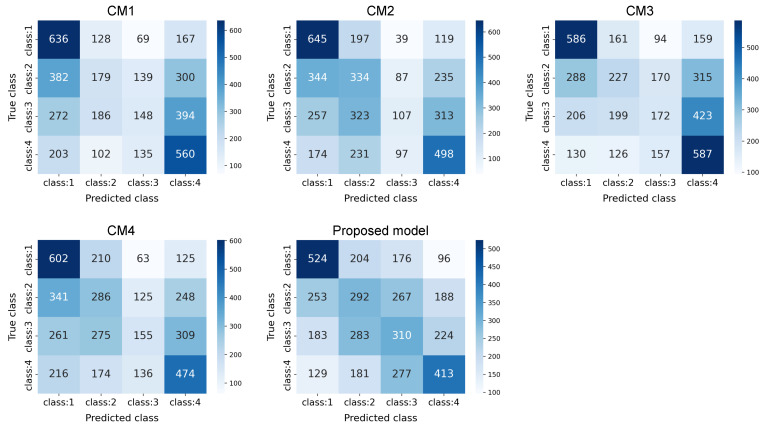
Confusion matrices of classification results based on the proposed and comparative models. Note that the horizontal and vertical axes represent the predicted and true classes, respectively.

Figure 5 shows the confusion matrices of classification results based on the proposed and comparative models. While the confusion matrices of the comparative models have high classification accuracy for “class:1”, they have low classification accuracy for the other classes. For example, in the results for CM1, the percentages classified as “class:2” and “class:3” are extremely low. Moreover, the confusion matrix of the proposed model has the largest diagonal component in each row. In other words, the proposed method confirms that the ratio of correctly classified classes exceeds the ratio of incorrectly classified classes. Furthermore, in this validation, since the class classification is based on the engagement rate separated by a certain range, there is an ordering among the classes. In the confusion matrix of the proposed model, adjacent elements of the diagonal elements also have higher values, which demonstrates the preservation of its ordering. These results also show that the proposed model can effectively classify the classes for the engagement rate compared to the comparative models.

## 5. Experiments

In this section, we validate the effectiveness of the proposed method that performs text-guided image editing based on post scores for gaining attention on social media. We compare the proposed method constructed based on the results in the previous Section 4 with state-of-the-art text-guided image editing methods. Section 5.1 and Section 5.2 explain experimental settings and results, respectively. Then, we discuss the effectiveness of the proposed method in Section 5.3.

### 5.1. Experimental Settings

In the proposed method, we applied the text-guided image editing method [15] pre-trained on a multi-modal training dataset [59] and generated four edited images for one input image. The inference time of the proposed method is mostly occupied by the time to generate the four edited images, which is approximately 40 s for one input image. The settings of the proposed model to predict post scores follow them in Section 4.1.

As comparative methods, we applied state-of-the-art text-guided image editing methods, namely, CLIPstyler [60], DiffEdit [61], and InstructPix2Pix [15]. While these methods achieve high accuracy in text-guided image editing, they are designed without considering the attention of the audience on social media for the edited images. Moreover, the proposed method generates edited images considering the attention of the audience on social media by newly introducing the model to predict post scores. By comparing the proposed method with these comparative methods, we verify the practicality of text-guided image editing, which even considers the way to utilize the edited images.

To verify the effectiveness of the proposed method, we conducted a subjective experiment. Specifically, in this experiment, we randomly selected 30 Instagram posts from the dataset divided into test sets in Table 1. Then, we created text prompts to perform text-guided image editing (e.g., “Summer”). Finally, to create samples evaluated by subjects, we applied the proposed and three comparative methods and obtained 120 (4 methods × 30 samples) edited images with the selected posted images and created text prompts. In the subject experiment, the following three perspectives were defined to evaluate the edited images by the subjects.

- Editing: the extent to which the edited image is accurately edited based on the text prompt.
- Response: the extent to which you would like to give it a “like” or comment when you find a post containing the edited image on social media.
- Aesthetics: the extent to which the edited image is aesthetic, where the accuracy of the editing is not considered.

As a subject experiment, we displayed the edited images generated by the proposed and comparative methods to 30 subjects who consented to participate in this experiment. Then, we asked these subjects to assign scores of 1–5 (1: worst, …, 5: best) to each edited image according to these three perspectives.

### 5.2. Accuracy of Proposed Method Compared to State-of-the-Art Methods

#### 5.2.1. Quantitative Results

Table 3 shows the results of Editing, Response, and Aesthetics obtained in the subjective experiment. Note that the values of these three perspectives are the mean and standard deviation for each text-guided image editing method, which were calculated based on the scores given by the subjects. These results demonstrate the effectiveness of the proposed method, which performs text-guided image editing considering attention on social media. Specifically, from the results of Editing, it can be seen that the proposed method achieves better accuracy of text-guided image editing compared with state-of-the-art methods. This means that the performance of text-guided image editing was not reduced by the consideration of attention on social media. The results of Response suggest that the edited images generated from the proposed method tend to gain attention from a greater audience on social media. Here, attention means that the posts containing the edited images receive many “likes” and comments. Furthermore, from the results of Aesthetics, it is demonstrated that the proposed method can generate the most aesthetic edited image. Since aesthetics have been elucidated to influence engagement rates [23], the results of Aesthetics in addition to them of Response further guarantee the effectiveness of the proposed method to generate edited images gaining attention on social media. We also conducted a Welch’s *t*-test on the proposed method and InstructPix2Pix which achieves second-best results and verified statistically significant differences of 1% (*p*-values < 0.01) in terms of all perspectives.

#### 5.2.2. Qualitative Results

**Figure 6:**
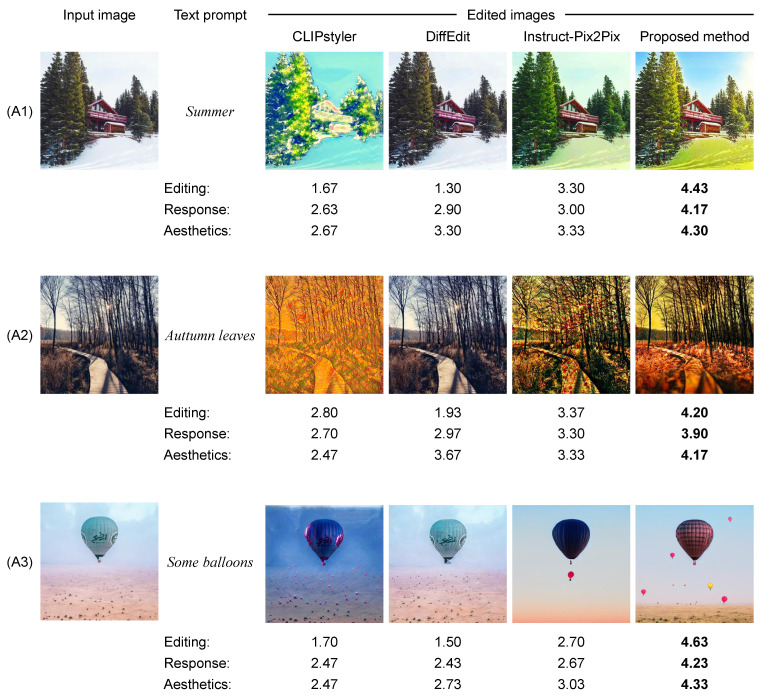
Qualitative results of the proposed and comparative methods [15,60,61]. The values under each edited image are Editing, Response, and Aesthetics obtained in the subject experiments. A bold value indicates the best result for each perspective. Symbols A1–A3 are referred to when discussing these results. Note that these images were posted by the usernames @derek_j, @alikirbymn, and @matteoacitelli on Instagram, which are included in the Instagram influencer dataset [18].

To evaluate the visual quality of the edited images, we compare the results obtained from the proposed and comparative methods in Figure 6. Below each edited image are the values for the three perspectives obtained in the subject experiments. The edited images generated from CLIPstyler are inferior to those of other methods in terms of performance of text-guided image editing (i.e., Editing). CLIPstyler is the method to perform style transfer based on a text prompt and is not designed to edit only text-related regions. Because of the design of CLIPstyler, it can be seen that there are limitations in editing the image in accordance with the text prompt while preserving text-unrelated regions. Also, it is suggested that the change of the entire image leads to an unnatural edited image, which also reduces the aesthetic and attention on social media. The edited images generated from DiffEdit are extremely similar to the input image, which demonstrates the limitations in editing images in accordance with the text prompt. While InstructPix2Pix achieves high accuracy in text-guided image editing compared to the other two comparative methods, it potentially generates edited images that are less likely to gain attention on social media. For example, in sample (A2), the edited image reflects the attribute of “Autumn leaves,” but with lower Aesthetics. This is supported by the values of Aesthetics obtained in the subject experiments. As a result, it is difficult to gain attention from a greater audience on social media.

In all samples, the proposed method successfully performs text-guided image editing while considering attention on social media. For example, in sample (A1), the edited image generated from the proposed method reflects the text prompt of “Summer” and has gained the most attention from the results of the Response perspective obtained from the subject experiment. Furthermore, the results of sample (A3) demonstrate that the proposed method can generate some colorful balloons into the edited image, which tends to gain attention on social media. This claim is guaranteed by the results of the perspectives of Response and Aesthetics.

### 5.3. Discussion

#### 5.3.1. Analysis of Model to Predict Post Scores

**Figure 7:**
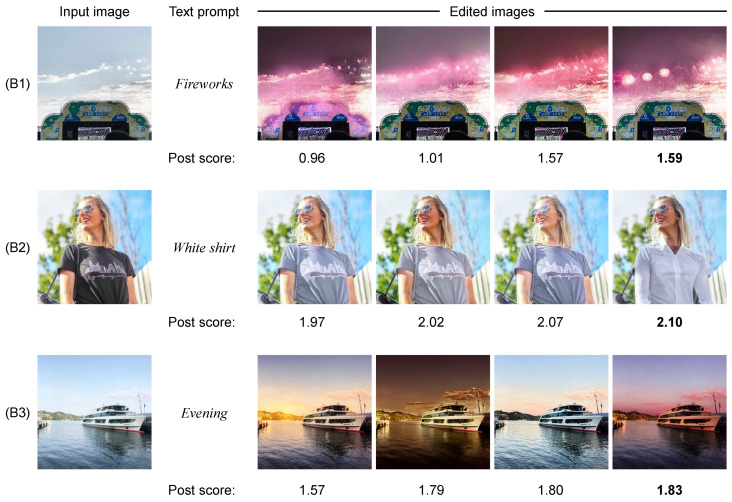
Visualization of the post scores assigned to the four edited images in the proposed method. The edited image with the highest post score is selected as the result of the proposed method. A bold value indicates the highest post score. Symbols B1–B3 are referred to when discussing these results. Note that these images were posted by the usernames @ruanbarreto, @lucyc0le, and @nataliawohler on Instagram, which are included in the Instagram influencer dataset [18].

Figure 7 shows the four edited images generated from multiple texts and the post scores assigned to them based on the proposed model. In sample (B1), although all the edited images have the attribute of “Fireworks,” they are represented in different ways. The leftmost edited image is less visible as a firework and is given the lowest post score. Moreover, it is easy to recognize fireworks in the rightmost edited image, and the highest post score is given to it. The results of sample (B2) demonstrate that the edited image given the highest post score correctly reflects the content of the text prompt, as well as has the most favorable impression compared to other edited images containing blurred-colored clothing. In sample (B3), the several edited images reflect the content of the text prompt. Among them, the edited image with the highest post score represents the magic hour (the time just after sunset and just before sunrise, producing warm colors of gold, pink, and blue [62]), which is generally considered to be the most fantastic moment of the evening hours.

#### 5.3.2. Limitations and Future Works

**Figure 8:**
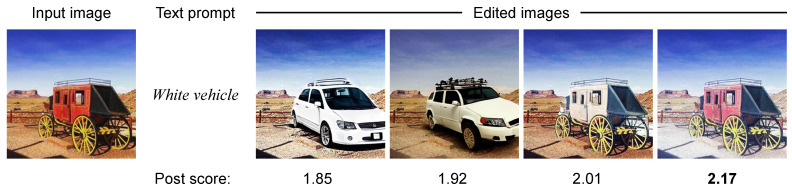
An example of the failure case based on the proposed method. The proposed method has the potential to give the highest post score described as a bold value to the insufficiently edited image. Note that the image was posted by the username @teronya on Instagram, which is included in the Instagram influencer dataset [18].

Although the proposed method successfully performed text-guided image editing while considering attention on social media, there are some cases where image editing is insufficient. The proposed method calculates post scores for four edited images generated based on the pre-trained text-guided image editing method and selects the edited image with the highest post score. That is, the proposed method works well under the condition that the four edited images are performed with highly accurate text-guided image editing. As shown in Figure 8, when four edited images contain ones performed insufficient text-guided image editing, the proposed method has the potential to give the highest post score to the insufficiently edited image.

In future works, the accuracy of image editing can be ensured by selecting edited images based on scores calculated from the evaluation metrics of text-guided image editing in addition to post scores. Also, since the accuracy of the proposed model to predict the post score was about 40%, we aim to improve its accuracy by devising a new integration technique for the image and text features. While the subject experiments demonstrated the effectiveness of the proposed method, we have not conducted a demonstration experiment in an actual environment of social media. Furthermore, we plan to verify the robustness of the proposed method by actually operating the accounts on social media.

## 6. Conclusions

This paper has proposed a novel text-guided image editing method based on post scores for gaining attention on social media. The proposed method newly introduces the model to predict post scores on social media from posted images and text, thereby generating edited images that gain much attention from the audience. In the proposed method, we apply the pre-trained text-guided image editing method and obtain multiple edited images from the multiple text prompts generated from the large language model. Among these, leveraging the novel model that predicts post scores representing engagement rates, the proposed method selects the edited images that will gain the most attention from the audience on social media. Results of subjective experiments demonstrated that the edited images generated from the proposed method accurately reflect the content of the text prompts and provide a positive impression to the audience on social media. These results are supported by the subjective evaluation that subjects are most willing to give a “like” or comment when they find posts including edited images generated from the proposed method on social media.

In the future, we will improve the proposed model to predict the post score by devising a new integration technique for the image and text features. Also, the accuracy of image editing can be ensured by selecting edited images based on scores calculated from the evaluation metrics of text-guided image editing in addition to post scores, enhancing the overall performance of the proposed method. 

## Figures and Tables

**Table 1:** Detailed statistics for the dataset extracted from the Instagram influencer dataset [18]. Note that the value of “train:validation:test” represents the split ratio.

	class:1	class:2	class:3	class:4
Number of data	10,000	10,000	10,000	10,000
train:validation:test	80%:10%:10%	80%:10%:10%	80%:10%:10%	80%:10%:10%
engagement rate	0.000~0.025	0.025~0.050	0.050~0.075	0.075~0.100

**Table 2:** Accuracy and F-measure of the proposed and comparative models in predicting classes for engagement rates. Bold values indicate the best results.

	Accuracy	F-Measure
CM1	0.380	0.381
CM2	0.396	0.396
CM3	0.393	0.393
CM4	0.379	0.379
Proposed model	**0.409**	**0.409 **

**Table 3:** The results of Editing, Response, and Aesthetics obtained in the subjective experiment. Note that the value is the mean and standard deviation of the evaluation values given by the subject for an edited image. Bold values indicate the best results.

	Editing	Response	Aesthetics
CLIPstyler	2.07±1.28	2.25±1.34	2.11±1.30
DiffEdit	1.79±1.30	2.64±1.24	2.98±1.27
InstructPix2Pix	3.20±1.41	3.00±1.28	3.06±1.28
Proposed method	4.17±1.08	3.56±1.18	3.83±1.13

## Data Availability

A publicly available dataset was used in this work. The dataset can be found here: https://sites.google.com/site/sbkimcv/dataset/instagram-influencer-dataset (accessed on 20 November 2023). Note that the dataset can be requested here: https://docs.google.com/forms/d/1KBgy1oj-Pf3g187yQxIvzRwq6td0sgmGONS5058Flyc/viewform?edit_requested=true (accessed on 20 November 2023).
